# Spatio-Temporal Regulation of Rac1 Mobility by Actin Islands

**DOI:** 10.1371/journal.pone.0143753

**Published:** 2015-11-25

**Authors:** Vinal V. Lakhani, Elizabeth Hinde, Enrico Gratton, Timothy C. Elston

**Affiliations:** 1 Curriculum in Bioinformatics and Computational Biology, University of North Carolina at Chapel Hill, Chapel Hill, North Carolina, United States of America; 2 Molecular and Cellular Biophysics Program, University of North Carolina at Chapel Hill, Chapel Hill, North Carolina, United States of America; 3 Department of Pharmacology, University of North Carolina at Chapel Hill, Chapel Hill, North Carolina, United States of America; 4 Australian Centre for NanoMedicine, University of New South Wales, Sydney, New South Wales, Australia; 5 Department of Developmental and Cell Biology, University of California Irvine, Irvine, California, United States of America; The University of Akron, UNITED STATES

## Abstract

Rho GTPases play important roles in many aspects of cell migration, including polarity establishment and organizing actin cytoskeleton. In particular, the Rho GTPase Rac1 has been associated with the generation of protrusions at leading edge of migrating cells. Previously we showed the mobility of Rac1 molecules is not uniform throughout a migrating cell (Hinde E et. al. PNAS 2013). Specifically, the closer a Rac1 molecule is to the leading edge, the slower the molecule diffuses. Because actin-bound Rac1 diffuses slower than unbound Rac1, we hypothesized that regions of high actin concentration, called “actin islands”, act as diffusive traps and are responsible for the non-uniform diffusion observed *in vivo*. Here, *in silico* model simulations demonstrate that equally spaced actin islands can regulate the time scale for Rac1 diffusion in a manner consistent with data from live-cell imaging experiments. Additionally, we find this mechanism is robust; different patterns of Rac1 mobility can be achieved by changing the actin islands’ positions or their affinity for Rac1.

## Introduction

Rho GTPases play a critical role in regulating many aspects of cell migration including polarity establishment and the actin cytoskeleton. Rac1 is a Rho GTPase associated with membrane protrusions at the leading edge of the cell [1]. Recent work demonstrated that Rac1 activity is closely regulated in space and time during the retraction portion of the protrusion-retraction cycle. Specifically, Rac1 activity peaks 40 seconds after and 2 μm away from a protrusion event [2]. Previously, we used fluctuation analysis in polarized cells to establish that the time scale of Rac1 diffusion varied with its localization within the cell [3]. Using pair correlation function (pCF) analysis, we calculated the time taken for a Rac1 molecule to move 1μm at each position along the axis of the cell [3]. In particular, we found a negative correlation between Rac1’s mobility and its proximity to the leading cell edge; Rac1 molecules took 100 times longer at the front of the cell than at the back to move 1μm [3]. We hypothesized that diffusive barriers, such as the ones found in neurons for compartmentalizing proteins [4], are responsible for the observed spatial variation in diffusion. Here we use a computational model to demonstrate that diffusive barriers, in the form of “actin islands”, can establish gradients of molecular mobility across the cell similar to those observed for Rac1.

We use a new technique called pair correlation function (pCF) analysis [5,6] to determine the spatial dependence of Rac1 mobility along the axis of a polarized cell. *In vivo* data were collected using a combination of Forster Resonance Energy Transfer (FRET) and Fluorescence Lifetime Imaging Microscopy (FLIM) [3,6]. We performed confocal line scans across the axis of a cell expressing a Rac1 dual chain FRET biosensor. The intensity and lifetime data of the donor and acceptor chain of this construct were collected by FLIM. This mode of acquisition provides us with two important data sets. First, we obtain a time series of the FRET biosensor lifetime in each pixel along the line scan, which describes the spatial distribution of Rac1 activity along the axis of the cell with millisecond resolution. Second, we obtain intensity fluctuations of Rac1 localization in each pixel along the line, which is used for pairwise correlation analysis of molecular flow along the axis of the cell. That is, we can calculate the time Rac1 molecules take to traverse a fixed distance along the line [3,6]. As noted above, using this multiplexed approach we recently found that Rac1 mobility decreases near the leading edge of the cell where we also observe, by FRET analysis, Rac1 activity to be the highest [3]. We hypothesized that cells achieve this spatiotemporal control of Rac1 mobility by using patches of dense actin, we call “actin islands”, to which Rac1 reversibly binds. By strategically placing and adjusting the density of the actin in these actin islands, the cell can reduce mobility of Rac1 in the desired location. For example, to slow diffusion towards the leading edge, the actin islands can be denser towards the leading edge.

**Fig 1:**
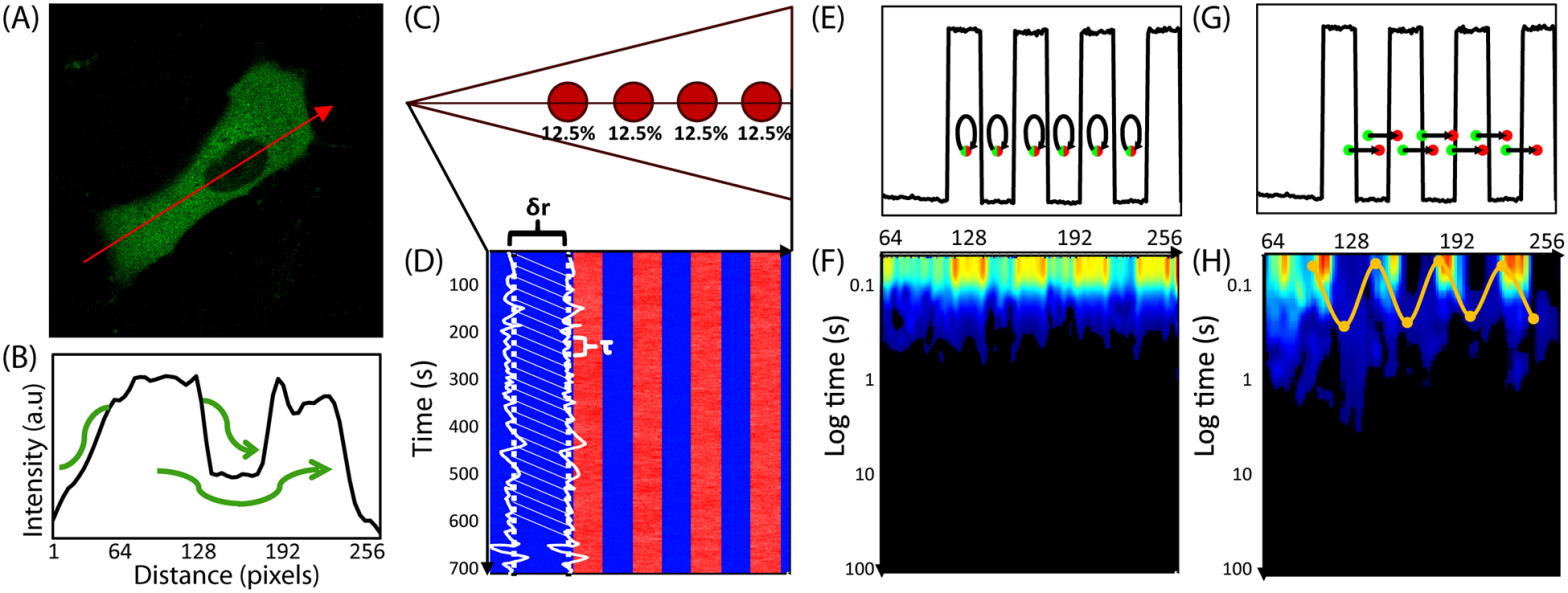
Pair correlation analysis of a fluorescent protein’s diffusive route reveals how the cell’s architecture directs intracellular traffic. (A) Intensity image of cell expressing 5-EGFP. Fluorescence data along the arrow is summarized in the next panel. (B) Intensity profile of 5-EGFP across axis of cell shows 5-EGFP exclusion from the nucleus and therefore an obstacle to 5-EGFP free diffusion. Green arrows demonstrate the molecules must diffuse around instead of through the obstacle. (C) Model for the simulation of Rac1 diffusion in a triangular shaped cell (25.6μm by 5μm) with four, circular traps (2μm wide). Each trap captures, on average, 12.5% of the total Rac1 population. Line scans are taken along the center axis of the cell, as shown by the horizontal line. Each pixel is measured in succession, and one line scan is completed when all 256 pixels have been measured. (D) Many (47000) line scans are combined into an intensity carpet. For this simulation, the intensity carpet shows accumulation of Rac1 colocalized with the four traps. A cartoon of two intensity profiles (intensity vs time) in white is overlaid on the intensity carpet. (E) A representative line scan (intensity vs pixel). The arrows indicate which two pixels are being pair-correlated, from green to red, for the pCF analysis in the next panel. In this case, each pixel is pair-correlated with itself, which is equivalent to an autocorrelation calculation. (F) The pCF(0) carpet reveals that pixels within the traps have higher autocorrelation values for short delay times (τ ≈ 0s) than pixels outside the traps. These values indicate the traps have a higher concentration of Rac1 than elsewhere in the cell. (G) The same representative line scan (intensity vs pixel) as in (E). Here the arrows indicate each pixel (green dot) is pair-correlated with a pixel (red dot) 0.5μm to the right (δr = 5 pixels). (H) The pCF(5) carpet reveals diffusion in and around the traps is slower than elsewhere in the cell. We average the data from every 20 pixels (columns) and smooth this average profile using a Gaussian filter; lastly, we extract the peak time for every 20 columns. We plot a point at each of these peak times. Hence, the yellow highlighted data displays the average time Rac1 takes to diffuse 0.5μm to the right. It takes about 0.3s to diffuse 0.5μm inside the islands but less than 0.1s to diffuse the same distance elsewhere in the cell.

To test this hypothesis, we created a computational model to study Rac1 mobility within a cell containing actin-islands. Using a particle-based stochastic simulation algorithm, we explicitly simulate the diffusion of individual Rac1 molecules and their binding/unbinding reactions with actin-islands. Unbound Rac1 freely diffuses throughout the cell. The actin-islands behave as diffusive traps, capable of slowing the diffusion rate and restricting the accessible space for an actin-bound Rac1 molecule. During the simulation we tally the number of Rac1 molecules located in bins along the center axis of the cell. Analogously, in the *in vivo* experiments, we measured the fluorescence intensity of pixels along the axis of the cell. In both cases, we tabulate the molecular counts (or fluorescence intensities) for each bin (or pixel) over time into an “intensity carpet” (Fig 1D). We use the intensity carpet to calculate a pCF carpet (Fig 1H)HHHHALSKDJFA;LSDJK as described in [3]. Although we model Rac1 diffusion as an unbiased, uniform process throughout the cell, we find the presence of actin-islands can create a spatial bias to Rac1 mobility. Hence, comparing the pCF carpet data, we qualitatively reproduce the *in vivo* data with our *in silico* model. These results give credence to our hypothesis that a mechanism based on strategically arranging actin islands can lead to spatially dependent molecular mobility.

## Results

### Intracellular Traffic Observed in *in silico* simulations by Pair Correlation Analysis

In live cells the default mechanism of motion for many biological molecules is diffusion. Although unregulated diffusion produces a spatially isotropic distribution of molecules, it is has been shown that structural features of the cell create intracellular compartments that generate spatially heterogeneous molecule distributions. For example, insights into intra-cellular trafficking have been derived from measuring the effect of the cell nucleus on the diffusion of biologically active and inert molecules [7]. The effect of the nucleus on diffusion is readily apparent, if we scan across the axis of an NIH3T3 cell transiently transfected with the biologically inert fluorescent protein 5-EGFP (Fig 1A). In this case, the fluorescence intensity profile clearly shows the exclusion of 5-EGFP from the nucleus (Fig 1B). From this simple experiment we can deduce that the nuclear envelope behaves as an impenetrable barrier, around which 5-EGFP must diffuse. Given that intracellular trafficking of biologically active molecules is far more complex, the diffusive route traveled by a fluorescently labeled protein is not always evident from simple inspection of the fluorescence intensity distribution, and thus a more dynamic approach is required.

To gain insight into the diffusive motion active proteins, we employ an analysis method that is based on pairwise correlation functions. Using pairwise correlation analysis, it is possible to discern both diffusion rates and particle fluxes along a confocal line scan. These quantities are inferred by measuring temporal cross-correlations in fluorescence intensity between pairs of points a distance δr apart as a function of the time delay τ between measurements. To illustrate this idea consider the following *in silico* example. We simulate the diffusion of individual Rac1 molecules (D = 10μm^2^/s) inside a cell containing four diffusive traps (Fig 1C). The cell is 25.6μm long and 5μm wide; the circular traps are 2μm in diameter (see Methods). Rac1 molecules that diffuse into a trapping area can reversibly bind to the trap. When bound in a trap, Rac1 molecules are (1) spatially restricted to remain inside the trap, and (2) the diffusion constant reduces to 1μm^2^/s. During the simulation we repeatedly take “line scans”, which are measurements along a line that traverses the cell (Fig 1C). Whereas the line scans for the *in vivo* experiments measured fluorescence intensity in each pixel (0.1μm)^2^ along the line, the line scans for our *in silico* simulations measure the number of molecules in square bins (0.1μm)^2^ along the line. In both cases, we summarize the resulting data as an intensity carpet (Fig 1D); wherein, each row is a single line scan (Fig 1E and 1G) and each column gives an intensity profile (intensity vs time) for a single pixel (Fig 1D).

The impact on mobility can be measured by pairwise correlation analysis between an intensity profile and a neighboring profile, a distance δr to the right, as a function of the time delay τ. We choose δr such that it is large enough to measure mobility around each trap. For example, we can calculate the correlation between the intensity profile at pixel 64 and the intensity profile at pixel 69 (δr = 5 or 0.5μm) τ seconds later. The characteristic delay time, defined as the τ that generates the highest correlation value, is a measure of the time scale for a Rac1 molecule at pixel 64 to diffuse to pixel 69. We repeat this process for all pixels to map the molecular flow pattern of Rac1 along the simulated cell’s axis. We first set δr = 0 and thus derive an autocorrelation profile (pCF(0)) for each pixel (Fig 1E and 1F). For τ = 0, the value of the autocorrelation is equal to the mean squared number of particles in the pixel. Hence, the high value areas in the pCF(0) carpet (Fig 1F) indicate areas of high Rac1 concentration. Unsurprisingly, these areas are co-localized with the actin island traps.

We next introduce a spatial component to the cross correlation function by setting δr = 5 pixels (pCF(5)) and recalculating the pCF carpet (Fig 1G and 1H). Taking δr = 5 pixels, corresponds to a distance of 0.5μm. This distance allows us to cross correlate intensity fluctuations located outside the trap with intensity fluctuations located inside the trap, thus measuring the time taken to enter or exit this environment. To help interpret the pCF carpet, we use the SimFCS software developed at the Laboratory for Fluorescence Dynamics (www.lfd.uci.edu); more details can be found in S1 Text as well as the literature [3,5,7]. Briefly, we combine and average every 20 pixels (columns) of the pair correlation values. Each average is smoothed with a Gaussian filter, and we highlight the peak times. Each peak time is the delay time with the maximum pair correlation value (Fig 1H). The highlighted points, plotted every 20 pixels, are connected by interpolation and indicate the time scale for Rac1 to diffuse 0.5μm to the right. We find Rac1 takes longer to diffuse in and around the actin islands than elsewhere in the cell. It takes about 0.3s to diffuse 0.5μm to the right inside the islands, which is consistent with the diffusional time scale of the islands: $\frac{L^{2}}{D}\;=\;\frac{{\left(0.5\mu m\right)}^{2}}{1\left(\mu m^{2}/s\right)}\;=\;0.25s$. Outside the islands, the same trajectory takes much less than 0.1s, which is as expected: $\frac{L^{2}}{D}\;=\;0.025s$. Hence, pCF analysis is capable of revealing that actin islands act as barriers to mobility.

### Gradient of Molecular Flow Observed in *in vivo* experiments by Pair Correlation Analysis

**Fig 2:**
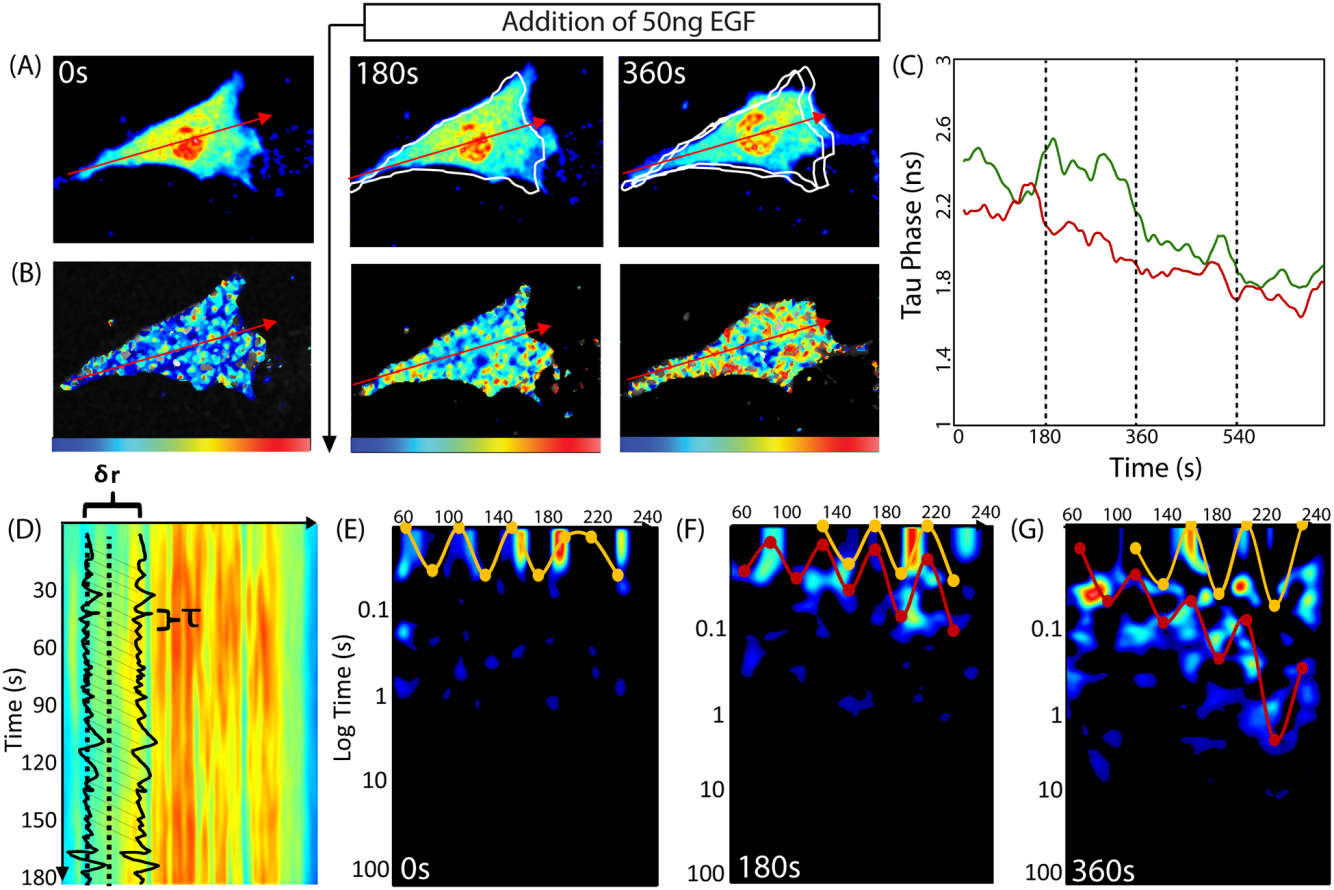
Pair correlation analysis of a Rac1 FRET biosensor reveals Rac1 activity to be spatiotemporally regulated by a dynamic gradient of protein mobility. (A) Intensity image of a NIH3T3 cell expressing the Rac1 dual chain FRET biosensor in the donor channel before and after stimulation with epidermal growth factor (EGF). The white traces outline the cell’s position(s) from the previous panel(s). (B) Same cell as in (A) pseudo-colored according to donor lifetime. The blue to red color range corresponds to a change in lifetime from 2 to 3ns and therefore low to high Rac1 activity. The tau phase (in ns) is derived from phasor analysis of the fluorescence decay, as in [3]. Shorter tau-phase times correspond to higher FRET activity. (C) Average lifetime analysis of the first 10 pixels (back of the cell, green time series) and the last 10 pixels (front of the cell, red time series). This comparison reveals that after EGF stimulation Rac1 is activated earlier at the front than the back of the cell. (D) The intensity carpet that is derived from line scans acquired across the axis of the cell in (A). (E) Pair correlation analysis of the intensity carpet acquired before EGF stimulation. The highlighted data shows Rac1 molecular flow is uniform: it takes about 0.03s to traverse 0.8μm (pCF(8)). (F) Pair correlation analysis of the intensity carpet acquired 180s after EGF stimulation. The highlighted shows Rac1 molecular flow is non-uniform. At the back of the cell, traversing 0.8μm takes about 0.03s; this time becomes gradually longer towards the front of the cell. The second set of highlighted data (yellow curve) is not significantly different from the first (red curve). (G) Pair correlation analysis of data acquired 360s after EGF stimulation. The mobility gradient is steeper (red curve); the delay time ranges from 0.05s at the back to 1.2s at the front. A second gradient emerges (yellow curve); the delay times range from 0.03s to 0.08s.

When probing the spatiotemporal dynamics of signaling molecules like Rac1, it is necessary to measure changes in both position and activity. These measurements are most often achieved by use of a FRET biosensor; wherein, changes in donor emission provide a readout of protein activity. Thus to determine the diffusive route Rac1 adopts upon activation, we recently combined biosensor FRET detection with pair correlation analysis [3]. Here, we repeat the experiment from [3]. We concomitantly measured the fluorescence intensity (Fig 2A) and fluorescence lifetime (Fig 2B) of a Rac1 biosensor in the donor channel along a line that extended from the rear to the front of a migrating cell. Lifetime analysis of the Rac1 biosensor FRET signal along the line scan revealed that the front of the cell (red time trace) is activated before the back of the cell (green time trace) (Fig 2C). In addition to this spatially dependent Rac1 activation, we found Rac1 mobility was also spatially dependent. We acquired three intensity carpets during the course of the experiment: one before EGF stimulation (Fig 2D), one between 0–180s after stimulation and one between 180–360s after stimulation. From each intensity carpet data, we performed pair correlation analysis of intensity fluctuations separated by a distance of 800nm (δr = 8 pixels) along the line scan (Fig 2E–2G). As in Fig 1H, we combined the data of every 20 columns into an average pair correlation vs delay time plot; we smooth this plot using a Gaussian filter. The highlighted red data points are the maxima of these smoothed curves (Fig 2E). For the data following EGF stimulation (Fig 2F and 2G), some of the smoothed curves have two maxima (see S1 Text for more details). The highlighted red and yellow data points are these maxima (Fig 2F and 2G). Before EGF stimulation (Fig 2E), Rac1 diffuses 800nm towards the front of the cell with a characteristic time of 0.03s. Note that this pCF carpet is qualitatively similar to the pCF carpet calculated from a simulation with uniform actin islands (Fig 1H). Three minutes after stimulation (Fig 2F), the time taken to travel 800nm is no longer constant across the axis of the cell (red curve Fig 2F); instead, there is a gradient of molecular flow with slower speeds near the front of the cell. After six minutes (Fig 2G), this gradient of flow is steeper (red curve Fig 2G); the characteristic times range from 0.1 to 1s. Additionally, the second set of highlighted data (yellow curve Fig 2G) is significantly different than the first, which indicates the presence of a second population of Rac1 whose mobility is spatially regulated separately from the first population.

The simulation in Fig 1 (Fig 1H), wherein each barrier has equivalent affinity for Rac1, qualitatively matches the mobility of Rac1 in an unstimulated cell (Fig 2E); that is, Rac1 mobility is uniform across the cell. However, in a stimulated cell, Rac1 shows variable mobility across the cell (Fig 2F and 2G), possibly due to variable density of actin and hence variable Rac1 binding affinity. It may be that Rac1 interacts with different substrates with varying affinities in the membrane, and therefore, Rac1 mobility depends on cell polarization in response to external cues. We next perform simulations guided by this hypothesis.

### Gradient of Rac1 Molecular Flow Produced by Actin Island Simulations

**Fig 3:**
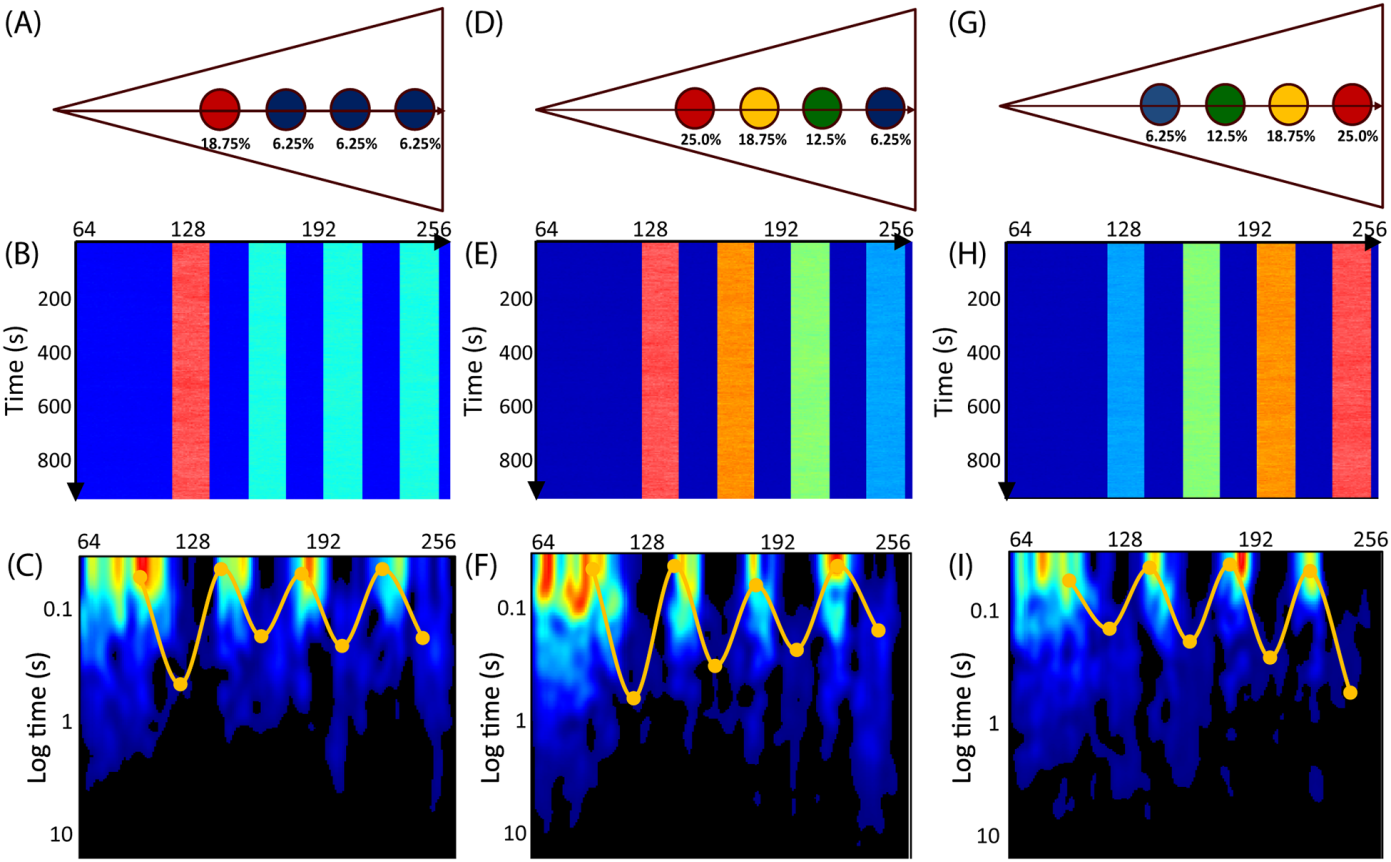
Simulations with islands of varying binding affinity. (A) A simulation set-up showing the rear (leftmost) island binds on average 18.75% of all Rac1, and each of the other three bind 6.25% on average. Unbound Rac1 diffuses with D = 10μm^2^/s. If bound, Rac1 diffuses with D = 1μm^2^/s. (B) The resulting intensity carpet shows the highest accumulation at the rear island. (C) The pCF carpet (yellow curve) reveals four arc features, which indicate regions of slow molecular flow, across each island. The time needed to flow 0.5μm to the right (pCF(5)) is longer near the island with the highest affinity (0.6s) than the other islands (0.2s). (D) A simulation wherein the islands form a gradient of binding affinities. The affinities range from 37.5–6.25% from the rear to the front of the cell. (E) The resulting intensity carpet shows a gradient of accumulation of Rac1. (F) The pCF carpet reveals a gradient of arc features whose position corresponds to the position of the islands and whose length correlates with the affinity of the islands. The time scale for molecular flow near the islands ranges from 1s, 0.8s, 0.5s and 0.2s (back to front). (G) Simulation of a cell with actin islands forming a gradient of binding affinities. The affinities range from 6.25–37.5% from the back to the front of the cell. (H) The resulting intensity carpet shows a gradient of accumulation of Rac1. (I) The pCF carpet reveals a gradient of arc features whose position corresponds to the position of the islands and whose length correlates with the affinity of the islands. The pair correlation pattern is opposite of the previous simulation (F). The time scale for molecular flow near the islands ranges from 0.2s, 0.5s, 0.8s and 1s (back to front). This gradient is analogous to the gradient calculated for 3min after EGF stimulation (Fig 2F).

To test our hypothesis that actin-islands can create the spatially dependent mobility observed from *in vivo* experiments, we returned to *in silico* simulations. Instead of assuming all the actin islands bind Rac1 with equal probability, we first tested a scenario in which the affinity of the rear island was taken to be three-times higher than the others (Fig 3A). The resulting intensity carpet showed four regions with higher intensity than the background, corresponding to the islands (Fig 3B). The region with highest intensity corresponds to the island with highest affinity for Rac1. Calculating the pCF carpet (Fig 3C) reveals that Rac1 molecular flow is slowest in the island with highest affinity, slightly faster in the other three islands and fastest outside the islands. Hence the islands’ affinity inversely correlates with local Rac1 mobility.

Next, we extend our model by varying the affinity of each actin island. Specifically, islands closer to the leading edge have lower affinity than islands near the trailing edge (Fig 3D). The intensity carpet shows four regions with different intensities (Fig 3E). As before, the average intensity in each of the four regions is directly proportional to the binding affinity. The pCF carpet (Fig 3F) shows a gradient of molecular flow; wherein, Rac1 mobility is faster towards the leading edge. We can reverse this gradient by reversing the island affinities (Fig 3G). This model produces a pCF carpet (Fig 3I) qualitatively similar to the one observed from *in vivo* data (Fig 2F). That is, there is a diffusive gradient such that Rac1 mobility is slower towards the leading edge. Consistent throughout all our simulations, we find the characteristic time to diffuse around or through an actin island is proportional to the island’s binding affinity. We find that this mechanism to spatially regulate molecular flow is quite robust; the affinity of the actin island dictates the mobility at that location.

**Fig 4:**
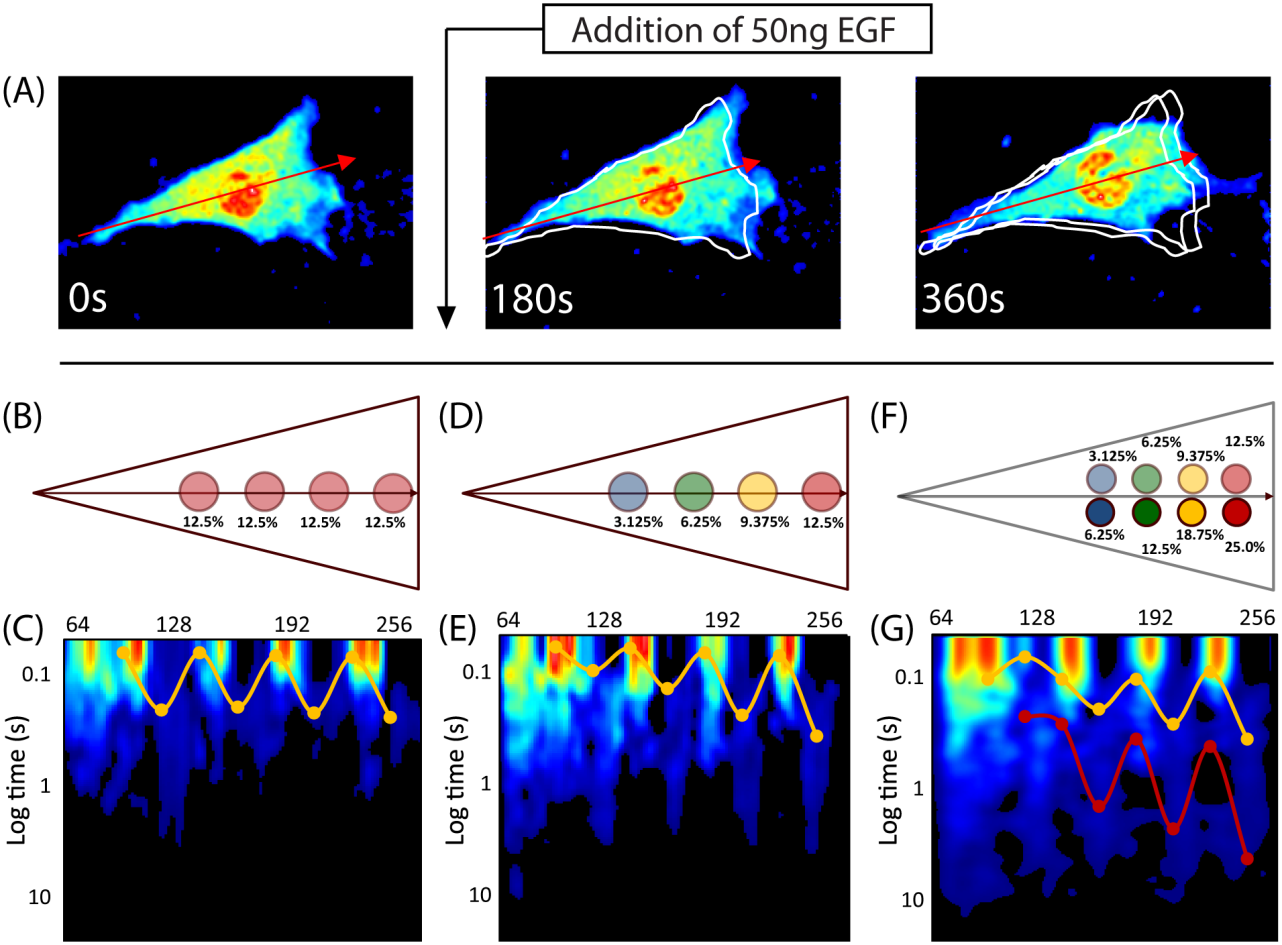
Investigating the cellular substructure during three stages of EGF stimulation by comparing pCF carpets. (A) Three snapshots of FLIM data before, 3 minutes after and 6 minutes after EGF stimulation. We aim to replicate features in the pCF carpets from the *in vivo* data (Fig 2E–2G) using our *in silico* simulations. (B—C) Uniform actin islands, as shown in Fig 1C and 1H. (D) Actin islands across the axis of the cell bind Rac1 with different affinities. These affinities range from 3.12% to 12.15% from the back to the front, which is less than the simulation in Fig 3G. This arrangement of islands is similar to what we expect is present *in vivo*. (E) pCF analysis reveals four arc features of differing lengths, co-localized with the actin islands. Rac1 mobility is slower towards the front of the cell. (F) Modeling two populations of Rac1 by the superposition of two actin island gradients: one with twice the affinity (Fig 3G) as the other (Fig 4D). We combine the intensity carpets of the two simulations and perform pCF analysis. Although the two sets of islands are superimposed, here, we separate them for illustrative purposes. (G) The resulting pCF carpet for a cell with two populations of Rac1. We find two distinct gradients as highlighted by the curves: one for each population. The red curve corresponds to the Rac1 population with the higher actin affinity.

Finally, we aimed to reproduce the molecular flow observed 6 minutes after EGF stimulation (Fig 2G), in which there are two populations of Rac1 whose mobility is regulated separately. It is known that active Rac1 moves slower than inactive Rac1 [8]. In our model, diffusion is regulated by Rac1’s affinity for actin (Rac1’s primary effector). We postulated that inactive Rac1 also binds actin, but with a significantly reduced affinity. Therefore, keeping all other parameters equal, we consider two populations of Rac1 molecules (inactive and active) that possess different affinities for the actin islands (Fig 4F). In particular, we assumed the active population binds actin with twice the affinity as the inactive population. For computational simplicity, we simulated each population separately (Figs 3G and 4D) and combined the resulting intensity carpets. This approach does not affect the results of our simulations, because the two subpopulations are assumed to diffuse and react independently of one another. After combining the intensity carpets, we perform pCF analysis. The resulting pCF carpet shows two gradients of molecular flow: one for each Rac1 population (Fig 4G). Based on our previous results, we know the red curve corresponds to the high affinity active Rac1 population, and the yellow curve corresponds to the low affinity active Rac1 population. Our results indicate actin islands, which reversibly bind Rac1 and slow its diffusion, are sufficient to produce the spatially dependent molecular flow observed *in vivo*. Additionally, we find that if active and inactive Rac1 have different affinities for actin, then these two subpopulations would show different diffusive behaviors, similar to what has been observed experimentally.

## Discussion

Here, we developed a computational platform for performing stochastic simulations of intracellular diffusion to study how actin islands are organized to spatially regulate the mobility of signaling molecules. Our simulation platform allows the location, shape, protein binding and unbinding rates and diffusion rate for each island to be varied independently. This flexible computational model allows us to probe what cellular architectures underlie key features of pCF carpets calculated from *in vivo* experiments using a Rac1 biosensor.

As proof of this principle, we considered diffusive regulation of Rac1 at three stages during EGF stimulation (Fig 4A). First, before stimulation, the molecular flow of Rac1 (assessed by the time needed for a Rac1 molecule to diffuse 0.5μm) is spatially uniform (Fig 2E). Second, for the first 3 minutes after stimulation, Rac1 mobility is inversely correlated with its proximity to the leading edge (Fig 2F). Third, between 3 and 6 minutes after stimulation, this gradient of molecular flow is more pronounced (red curve Fig 2G), and the presence of a distinct second set of arc features (yellow curve Fig 2G) indicates a second population of Rac1 that moves differently than the first. To test if the existence of actin islands can produce arc features in the pCF carpet consistent with our experimental observations, we computationally simulated the diffusion of Rac1 in a cell with islands of equivalent affinity (Figs 1H and 4C). The good qualitative agreement between the pCF carpets from *in vivo* (Fig 2F) and *in silico* (Fig 4C) experiments suggests actin islands are present before EGF stimulation (Fig 4A–4C). Next, to test our hypothesis that actin island affinity regulates molecular flow, we performed simulations with actin islands of varying affinity (Figs 3G and 4D) and compared these with the *in vivo* observed mobility gradient (Fig 2F). The pCF carpets from these simulations (Figs 3I and 4E) show a mobility gradient similar to the one observed *in vivo* (Fig 2F); wherein, the time it takes for Rac1 molecules to flow 0.5μm is based on their proximity to the leading edge. Hence, the observed mobility gradient from the *in vivo* experiment is consistent with the presence of actin islands with progressively stronger affinity for Rac1 in moving from the rear to the front of the cell. Finally, we aimed to reproduce the *in vivo* observed dual regulation of Rac1 indicated by two distinct mobility gradients (Fig 2G). We combine the results of two simulations, which are identical in every aspect, except for the actin islands’ affinities (Fig 4F). The resulting pCF carpet (Fig 4G) shows two sets of arc features, similar to the ones observed *in vivo* (Fig 2G). Hence, the two sets of mobility gradients observed from the *in vivo* experiments (Fig 2G) may indicate the presence of two forms of Rac1 (e.g. inactive and active) with different affinities to the actin islands.

Our results suggest the Rac1flow observed *in vivo* (Fig 2F and 2G) is produced when actin islands near the leading edge have a higher affinity than islands near the trailing edge (Fig 3G–3I). There are many possible explanations for this discrepancy in affinities. Because the affinity in an island is proportional to the actin concentration, one possibility is that the actin concentration is higher in islands near the leading edge than those near the trailing edge. Another possibility is that the conformation of Rac1 near the leading edge is different than Rac1 near the trailing edge; moreover, this difference alters the affinity of Rac1 to actin. Another Rac1 binding partner may account for the discrepancy in conformation sampling. We discuss the implications of each possible technique for controlling the affinity of actin islands below.

Based on the results of our experimental and computational investigations, we propose that cells can spatially regulate the molecular flow of certain proteins through the use of actin islands. In particular, our results suggest that cells can position the islands in regions where slower flow is desired, e.g. to sequester Rac1 at the leading edge. Because an island regulates molecular flow only locally, the cell can utilize actin islands only where needed. Additionally, the extent to which Rac1 mobility is slowed can be regulated by adjusting the binding affinity. Actin is known to reorganize in seconds [9], and this reorganization may play a role in regulating the position, size, shape and concentration of the islands. In turn, the concentration of actin in each island could affect the affinity for Rac1: denser islands have higher affinity than less dense islands. Organizing actin islands allows cells to spatially regulate molecular flow and therefore establish internal concentration gradients.

Another important feature is molecularly independent regulation; different protein species, and/or different forms of the same protein species, can be regulated separately. As long as each protein population has a different affinity for actin, each population will effectively experience a different set of actin islands (Fig 4F and 4G). We successfully tested a scenario (data not shown), wherein the two populations had opposing molecular flow gradients (i.e. a combination of Fig 3F and 3I) by combining opposing actin island gradients (i.e. Fig 3D and 3G). Actin islands can differentially regulate the diffusion of different proteins across the cell; furthermore, pCF carpet analysis is capable of distinguishing these different protein populations and their unique, spatial regulation of molecular flow. Hence, the two sets of features from the *in vivo* experiment (Fig 2G) indicate there are two populations of Rac1 (possibly the inactive and active forms) with different affinities for the actin islands and subsequently separately regulated flow.

## Methods

We developed a simulation platform to test our proposed model of Rac1 behavior. Our program returns intensity carpets that are directly comparable to experimentally measured intensity carpets. As with the *in vivo* experimental data, we perform pair correlation analysis on the *in silico* intensity carpets to determine any spatial dependence on the diffusion constant. Our goal was to determine sufficient conditions for recapitulating the spatial dependence on the diffusion constant observed *in vivo*, by rearranging and varying the binding constant of the actin islands. Our program uses a particle-based stochastic simulation algorithm to simulate reactions and diffusion. Individual Rac1 molecules are modeled as point particles in continuous space and capable of reacting and diffusing discretely in time. This algorithm allows us to closely mimic the stochastic diffusion of Rac1 molecules inside the cell. A computational model that captures these natural fluctuations in concentration is imperative for pair correlation analysis.

### Simulation Algorithm

**Fig 5:**
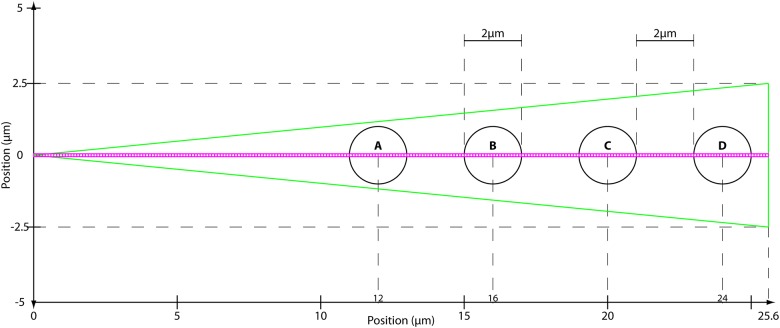
Simulation Geometry. The cell boundary is shown in green as an isosceles triangle 25.6μm wide and 5μm tall. The actin islands are shown as black circles with 1μm radii. As discussed in the text, unbound Rac1 molecules are free to diffuse throughout the cell and reflect off the cell boundary. Bound Rac1 molecules diffuse slower and are restricted to the inside of the island in which they are bound. We can individually set the K_D_ of each island thereby setting, on average, the percent of the total Rac1 population bound to each island. The 256 magenta boxes represent the (0.1μm)^2^ bins used to generate the intensity carpet; these bins do not affect the behavior of the molecules.

A cell migrating on a 2D substrate in the xy-plane is modeled as an isosceles triangle whose base represents the direction of migration (Fig 5). In this system, diffusion along the z-axis is largely inconsequential, because the molecular counts used to produce the intensity carpets *in vivo* are projected onto the xy-plane, thereby destroying any information from the third dimension. Additionally, the computational complexity is significantly reduced when only 2 dimensions are considered. Rac1 molecules are modeled as non-colliding point particles; their positions are stored in (x,y) Cartesian coordinates. Our hypothesized actin islands, regions of dense actin molecules, are capable of binding freely diffusing Rac1 molecules; hence, these actin islands behave like diffusive traps, since actin-bound Rac1 has a smaller diffusion constant. The actin islands are modeled as circular patches, which can reversibly bind any Rac1 molecules located inside these regions (Fig 5).

As suggested by [10], we use the Euler-Maruyama method [11] to perform stochastic simulations of diffusing particles. That is, knowing the current position of a Rac1 particle, (x(t), y(t)), we calculate its position at the next time step, (x(t+Δt),y(t+Δt)), using the following equations:

$$x\left(t+\Delta t\right)=x\left(t\right)+W_{i}\sqrt{2D\Delta t}$$ (1)

$$y\left(t+\Delta t\right)=y\left(t\right)+W_{j}\sqrt{2D\Delta t}$$ (2)

Here, *D* is the diffusion constant, *Δt* is the time step and the *W*_*n*_’s are random numbers drawn from a Gaussian distribution with a mean of 0 and a variance of 1. Unbound Rac1 particles diffuse freely inside the cell with D_unbound_ = 10μm^2^/s. The boundaries of the cell are reflective. Hence, if a molecule attempts to leave the cell (i.e. the diffusion calculation places the particle outside the cell boundaries), then the molecule is elastically reflected back inside the cell. Bound Rac1 particles diffuse freely inside the actin island to which they are bound with D_bound_ = 1μm^2^/s. The boundary of the island is reflective only to bound molecules. Hence, if a bound molecule attempts to leave the island (i.e. the diffusion calculation places the particle outside the island), then the molecule is elastically reflected back inside the island. Consequently, all bound Rac1 molecules are restricted to an island. The converse, however, is not true; not every Rac1 molecule positioned inside an actin island is bound. Unbound Rac1 molecules, which are positioned inside an island, are considered to be diffusing over/under/through that island without penalty. It is only in this condition that a binding reaction may occur.

In addition to diffusion, binding and unbinding events are also calculated at each time step. If a Rac1 molecule is, unbound and positioned inside an island at time *t*, then the state of that molecule can be switched to bound with probability: P_bind_ calculated for each island by:

$$P_{bind}\;=\;\left(\frac{v}{V}\right)\left(1\;-\;e^{-\frac{\Delta \text{t}}{v}\cdot k_{on}}\right)$$ (3)

Here, *k*_*on*_ is the rate constant for binding to the island; *Δt* is the time step; *v* is the volume of the island, and *V* is the volume of the entire cell (we assume the cell is 1μm thick).

Separately, we calculate all unbinding events between time steps. If a Rac1 molecule is bound at time *t*, then its state can switch to unbound with probability P_unbind_:

$$P_{unbind}=1-e^{-\Delta t\cdot k_{off}}$$ (4)

Here, k_off_ is the dissociation rate constant. When changing the state of a Rac1 molecule, due to either a binding or unbinding reaction, we do not change the position of the molecule.

We calculate the reaction rates based on an average fraction of total Rac1 molecules that we wish to have bound in each island Eq 5.

$$\frac{1}{V}\frac{k_{on}}{k_{off}}=\frac{f}{1-F}$$ (5)

Here, *f* is the average fraction of Rac1 molecules bound in the island, and *1–F* is the average fraction of Rac1 molecules not bound in any island. Note k_on_ should be expressed in units of μm^3^/s using a conversion factor such as 0.6022/(nM μm^3^). Additionally, the fraction of molecules bound at each island also relates to the reaction probabilities Eq 6.

$$\frac{v}{V}\frac{P_{bind}}{P_{unbind}}=\frac{f}{1-F}$$ (6)

For example, if we desire each of the four islands to bind, on average, 6.25% of all Rac1 molecules, then F = 0.25 and for each island *f* = 0.0625. We know the volumes (*v* and *V*) from Fig 5. Unfortunately, instead of a value for P_bind_ or P_unbind_, we are only left with a ratio between these reaction probabilities. We proceed by assigning an off-rate within the Biological regime. We choose the off-rate such that the time-scale for unbinding is equal to the time-scale for a bound molecule to diffuse the length of the island: see Eq 7.

$$\begin{array}{l} \Delta x=\sqrt{2D\Delta t}\\ k_{off}=\frac{1}{\Delta t}=\frac{2D_{bound}}{\Delta x^{2}}=\frac{2\left(1\frac{\mu m^{2}}{s}\right)}{{\left(2\mu m\right)}^{2}}=0.5\frac{1}{s} \end{array}$$ (7)

This rate is substituted into Eq 4 to get P_unbind_, which in turn, is substituted into Eq 6 to get P_bind_. In this case, P_unbind_ = 0.5×10^−6^ and P_bind_ = 0.85×10^−6^ for a time-step Δt = 1μs. The reaction probabilities for each simulation are annotated in S1 Text.

### Pair Correlation Carpet

A line-scan technique was used during *in vivo* experiments [3,5,6], which reports the fluorescence intensity of Rac1-GFP molecules. The intensity is sequentially measured from pixel 1 to, about, pixel 300 to complete one line scan. Many line scans are taken and these scans are compiled into an intensity carpet on which pair correlation analysis can be performed. To produce an *in silico* analog to the intensity carpet, we tally the particles by their position as they are simulated (see above). To establish the line along which we will scan, we arrange 256 square bins sized (0.1μm)^2^ along the center of the cell (Fig 5). We cumulatively count the number of Rac1 molecules positioned inside a bin during a 25μs time window. Each bin is scanned sequentially; that is, we tally the counts from the first bin for the first 25μs; then we tally the counts from the second bin for the second 25μs etc. After 6.4ms (256*25μs) one full line scan is complete, and the next line scan begins immediately. Each intensity carpet has 47000 *lines*, corresponding to a simulation time of just over 5min. The bin size (0.1μm)^2^ is roughly equivalent to the size of the laser used in the *in vivo* experiments, and the measuring time (25μs) is equivalent to the length of time a pixel was monitored *in vivo*.

To better understand the flow of particles within the cell, we calculate the pair correlation function (pCF) from the intensity carpet. First, we extract the intensity time trace (intensity versus time) for each pixel (e.g. cartooned white curves in Fig 1D). Second, we calculate the correlation coefficient between two of these traces while sweeping two parameters in space (δr) and time (τ). With regards to space, we consider the traces of two pixels a distance δr apart; note that when δr = 0, we are computing the autocorrelation value (Fig 1E and 1F). With regards to time, we shift the trace from the second pixel by τ seconds. Hence the correlation coefficient for a pixel given a pair of parameters (δr, τ) describes the likelihood a particle will diffuse from that pixel to a pixel δr away in τ time. A value of 0 indicates this diffusive trajectory is impossible; while, a value of 1 implies a complete directed flow. Note that there is directionality in our pCF. A left to right calculation (e.g. correlating pixel 0 with pixel 5) describes the diffusion landscape from the back of the cell to the front (line scans are taken as such). A right to left calculation (e.g. correlating pixel 5 with pixel 0) describes the diffusion landscape from the front of the cell to the back.

Our results are independent of the cell’s shape, because the cell boundary is sufficiently far from our region of interest. For example, the sharp, leftmost corner produces a slight force to the right; however, this force quickly dissipates and has little effect beyond x = 5μm. All the pCF carpets presented here show pCF analysis for x≥6.4μm.

## Supporting Information

S1 Dataset From Fig 1D. This Matlab file contains variables used to generate Fig 1D. The variable ‘LinesScanned’ is the number of lines scanned; ‘Npixels’ is contains the number pixels or bins, as shown in Fig 5; ‘t’ is an array of the time points for each line scan in units of seconds. The variable ‘IntCarpet’ is a matrix (‘LinesScanned’ rows by ‘Npixels’ columns); each element contains a tally of the molecules for that bin in that time frame. Consider the following three examples. Element (1,1) is the tally of molecules in bin 1 (located near x = 0μm) between t = 0s to 25μs. Element (1,2) is the tally of molecules in bin 2 (located near x = 0.1μm) between t = 25μs to 50μs. Element (2,1) is the tally in bin 1 between t = 6.4ms to 6.425ms.
(MAT)

S2 Dataset From Fig 3B. This Matlab file contains variables used to generate Fig 3B. See description in S1 Dataset for explanation of the variables in this file.
(MAT)

S3 Dataset From Fig 3E. This Matlab file contains variables used to generate Fig 3E. See description in S1 Dataset for explanation of the variables in this file.
(MAT)

S4 Dataset From Fig 3H. This Matlab file contains variables used to generate Fig 3H. See description in S1 Dataset for explanation of the variables in this file.
(MAT)

S5 Dataset From Fig 4D. This Matlab file contains variables used to generate Fig 4D. See description in S1 Dataset for explanation of the variables in this file.
(MAT)

S6 Dataset From Fig 4F. This Matlab file contains variables used to generate Fig 4F. See description in S1 Dataset for explanation of the variables in this file.
(MAT)

S1 Movie Simulation Example. This movie shows an example of a simulation with fewer molecules than those simulations used to generate the intensity carpets reported in the paper. Here, 1000 Rac1 molecules are plotted in a cell similar to Fig 4G; wherein, the actin islands increase in affinity from left to right. Each island, from left to right respectively, binds 3.125%, 6.25%, 9.375% and 12.5% of the total Rac1 population. To obtain statistically significant data, we simulate 100,000 Rac1 molecules to generate the intensity carpets reported above.
(MOV)

S1 Text Supplemental Methods. This text contains a detailed description of our simulation algorithm. We discuss how Rac1 molecules are reflected off the cell and actin island boundaries. We derive the reaction rates, reaction probabilities and list these parameters for each result figure presented in the paper. We discuss how the Intensity Carpets are generated. Finally, in this text we discuss some details on the computer hardware used for the simulations.
(DOCX)
