# Supplementary material for: Spatio-Temporal Regulation of Rac1 Mobility by Actin Islands
Source: PLoS One. 2015 Nov 25;10(11):e0143753. doi: 10.1371/journal.pone.0143753 (PMC4659588; doi:10.1371/journal.pone.0143753)
Supplement: S1 Text — This text contains a detailed description of our simulation algorithm. We discuss how Rac1 molecules are reflected off the cell and actin island boundaries. We derive the reaction rates, reaction probabilities and list these parameters for each result figure presented in the paper. We discuss how the Intensity Carpets are generated. Finally, in this text we discuss some details on the computer hardware used for the simulations. (DOCX) [file pone.0143753.s008.docx]

**Supplemental Methods**

**Analyzing pCF carpets**


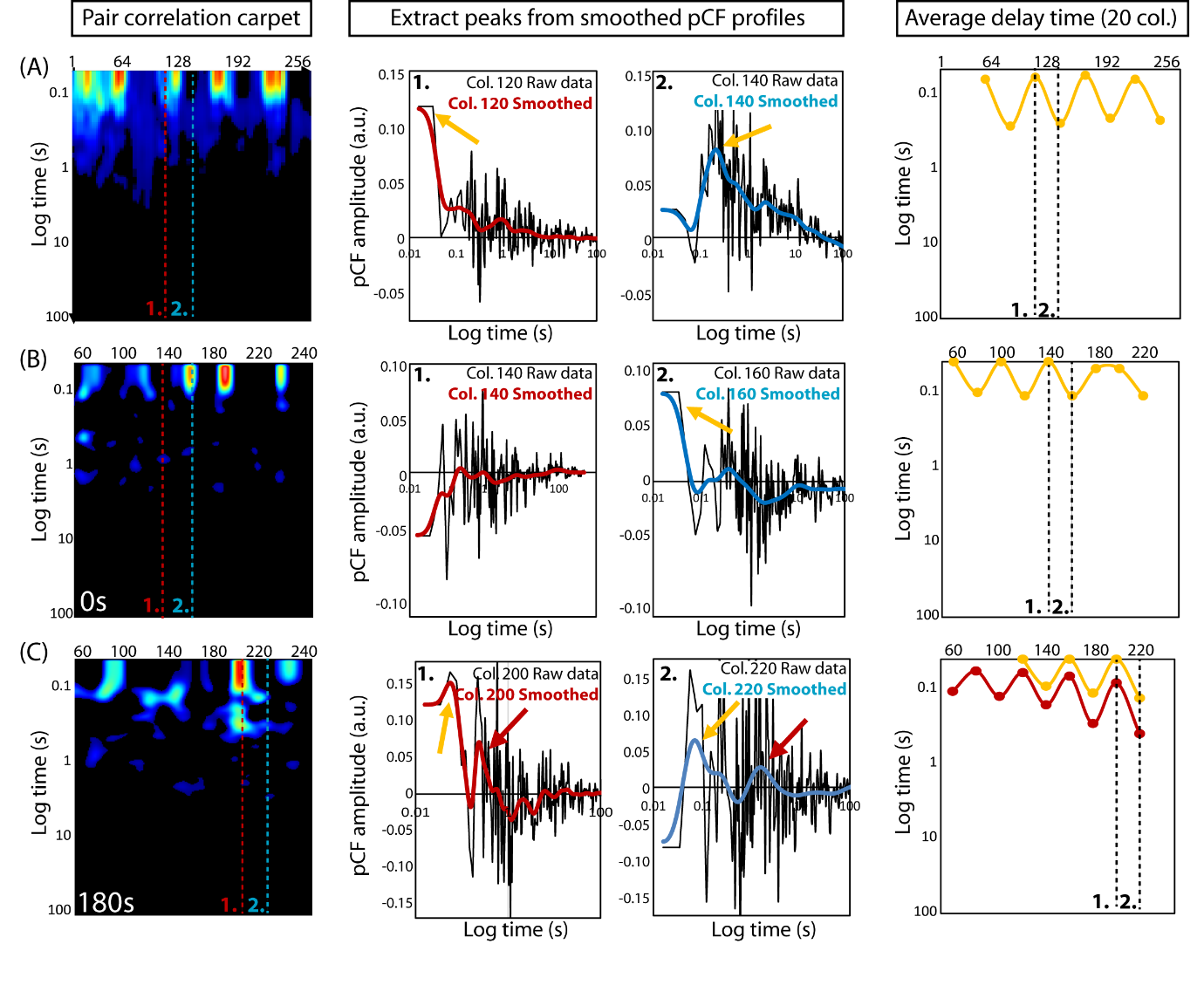


**Figure S1: Gaussian analysis of pair correlation carpets to extract average delay time(s) Rac1 takes to diffuse along simulated and *in vivo* line scans.**

We smoothed every column in the derived pair correlation carpets by use of a Gaussian filter and then extracted the average peak time(s) for every 20 columns to obtain a simplified temporal profile of the molecular flow occurring at each consecutive location along the line scan. We then plotted the extracted peak time(s) as a function of position along the line scan. (A) Pair correlation carpet presented in Fig. 1H and an example of how the Gaussian filter smooths the pair correlation profile at two columns (120 and 140). (B) Pair correlation carpet presented in Fig. 2E and an example of how the Gaussian filter smooths the pair correlation profile at two columns (140 and 160). (C) Pair correlation carpet presented in Fig. 2F and an example of how the Gaussian filter smooths the pair correlation profile at two columns (200 and 220). The analysis presented in (A)-(C) enabled us to extract the average time(s) for molecular flow every 20 columns from pixel 60-220.

**Simulation Program Details**

We simulate $100,000$ molecules, which, given the dimension of the cell (Fig 5) and an assumed height of 1 μM, corresponds to a concentration of $0.94 \mu M$. We monitor each molecule’s position: $\left( x,y \right)$ and its state: ‘bound’ or ‘unbound’. Box 1 presents pseudo-code for the general algorithm used to simulate Rac1 behavior in the cell.

Loop for **T** time steps

Loop for **M** molecules

if **m_i_** is ‘unbound’

Diffuse with $D_{unbound}=10\frac{{\mu m}^{2}}{s}$ (1)

$\left( x,y \right)_{t}\to\left( x,y \right)_{t+\Delta t}$ (2)

If $\left( x,y \right)_{t+\Delta t}$ is outside the cell, then reflect the position back inside cell (3)

if $\left( x,y \right)_{t+\Delta t}$ is inside actin island **a_j_** (4)

Draw a uniform random number between 0 and 1: $U_{[0,1)}$

if $U_{[0,1)}\leq P_{bind}$ for **a_j_** (5)

Set **m_i_** to ‘bound’ to island **a_j_**

else **m_i_** is ‘bound’ to island **a_j_**

Diffuse with $D_{bound}=1\frac{{\mu m}^{2}}{s}$ (6)

$\left( x,y \right)_{t}\to\left( x,y \right)_{t+\Delta t}$ (7)

If $\left( x,y \right)_{t+\Delta t}$ is outside island **a_j_**, then reflect the position back inside island **a_j_** (8)

Draw a random number between 0 and 1: $U_{[0,1)}$

if $U_{[0,1)}\leq P_{unbind}$ for **a_j_** (9)

Set **m_i_** to ‘unbound’

Finish updating all molecules

Finish Simulation

**Box 1: Simulation Outline**

All particles are updated (regarding diffusion and reaction) simultaneously during each time step. Depending on the state of the particle (‘bound’ or ‘unbound’), we calculate its new position (Diffusion) then determine if its state changes (Reaction). In memory, we store the position and state of each particle as well as the island to which it is bound. Important lines are numbered.

**Diffusion**

Particle Diffusion is simulated in lines (2) & (7) of Box 1. We use Eqns 1 & 2 as defined in the Methods to get the particles’ new position. That is, for each spatial dimension: $x\left( t+\Delta t \right)=x\left( t \right)+W\sqrt{2D\Delta t}$. Here, $W$ is a random number drawn from a Gaussian Distribution with mean 0 and variance 1. We calculate this random number using the Box-Muller Method, as implemented in the “curand” library of the CUDA 4.0 Toolkit.

As noted in lines (3) & (8) of Box 1, particles are restricted to certain regions. ‘Unbound’ particles must remain inside the cell, and ‘bound’ particles must remain inside the island to which they are bound. These rules are enforced by imposing reflective boundaries at the cell wall (Box 2) & edges of the actin islands (Box 3) respectively.

*Cell boundaries: Derivation*

Let $\left( x^{'},y' \right)$ be the position that lies outside the cell. The cell’s boundary can be described as three lines: the ‘top’ boundary traversing from $\left( 0, 0 \right)$ to $\left( 25.6, 2.5 \right)$, the ‘bottom’ boundary traversing from $\left( 0, 0 \right)$ to $\left( 25.6, -2.5 \right)$, and the ‘right’ boundary traversing from $\left( 25.6, -2.5 \right)$ to $\left( 25.6, 2.5 \right)$ (Fig 5). We describe each line in the form: $ax+by+c=0$ (restricting $a>0$). Accordingly, the distance from the point $\left( x^{'},y' \right)$ to a line can be calculated.

$$d=\frac{\left| ax^{'}+by^{'}+c \right|}{\sqrt{a^{2}+b^{2}}}$$

Hence, the point $\left( x^{'},y' \right)$ can be reflected off the ‘top’ and ‘bottom’ boundaries using the following equation.

$$\left[ \begin{matrix} x \\ y \end{matrix} \right]=\left[ \begin{matrix} x' \\ y' \end{matrix} \right]+\frac{2d}{\sqrt{a^{2}+b^{2}}} \left[ \begin{matrix} a \\ b \end{matrix} \right]$$

To reflect the point $\left( x^{'},y' \right)$ off the ‘right’ boundary, we calculate:

$$x=x^{'}-2*\left| x^{'}+\frac{c}{a} \right|$$

*Cell boundaries: Implementation*

bool **high** = $y^{'}> \left( \frac{5.0}{2*25.6} \right) x'$ (1)

bool **low** = $y^{'}< \left( \frac{-5.0}{2*25.6} \right) x'$ (2)

bool **right** = $x^{'}> 25.6$ (3)

for ( **i** = 0; ( **i** < 25 ) AND ( **high** OR **low** OR **right** ); **i** ++ ) (4)

if ( **high** ) (5)

$a=5 \mu m$ $b=-2*25.6 \mu m$ $c=0 \mu m$ (6)

$\left[ \begin{matrix} x' \\ y' \end{matrix} \right]=\left[ \begin{matrix} x' \\ y' \end{matrix} \right]+\frac{2*\left| \left( 5 \right)x^{'}-\left( 2*25.6 \right)y' \right|}{5^{2}+\left( -2*25.6 \right)^{2}} \left[ \begin{matrix} 5 \\ -2*25.6 \end{matrix} \right]$ (7)

else if ( **low** ) (8)

$a=5 \mu m$ $b=2*25.6 \mu m$ $c=0\mu m$ (9)

$\left[ \begin{matrix} x' \\ y' \end{matrix} \right]=\left[ \begin{matrix} x' \\ y' \end{matrix} \right]+\frac{2*\left| \left( 5 \right)x^{'}+\left( 2*25.6 \right)y' \right|}{5^{2}+\left( 2*25.6 \right)^{2}} \left[ \begin{matrix} 5 \\ 2*25.6 \end{matrix} \right]$ (10)

else if ( **right** ) (11)

$a=1 \mu m$ $b=0 \mu m$ $c=-25.6 \mu m$ (12)

$x^{'}=2*25.6-x'$ (13)

else {}

Re-check **high**, **low** and **right** (repeat lines 1 – 3)

end

**Box 2: Cell Boundary Reflection Calculations**

During the diffusion of an ‘unbound’ particle, we check if the new, proposed position is outside the cell. We check if the proposed position is too high: beyond the upper boundary (line 1), too low: beyond the lower boundary (line 2), and/or too far right: beyond the right boundary (line 3). If the particle is out of bounds, then we reflect the proposed position with respect to the appropriate boundary. For example, if the position is too high, then we reflect the position about the upper boundary. This method of checking and reflecting is repeated until all checks pass (i.e. the position is inside the cell) or 25 reflections have been made. This latter condition protects against pathogenic cases that lead to an infinite check/reflect loop. These rare cases occur when the position is close (within numerical error) to a corner of the cell boundary. These cases are resolved during diffusion in the next time step. This algorithm is an expansion of line 3 in Box 1.

*Actin Island boundaries: Visual Guide*


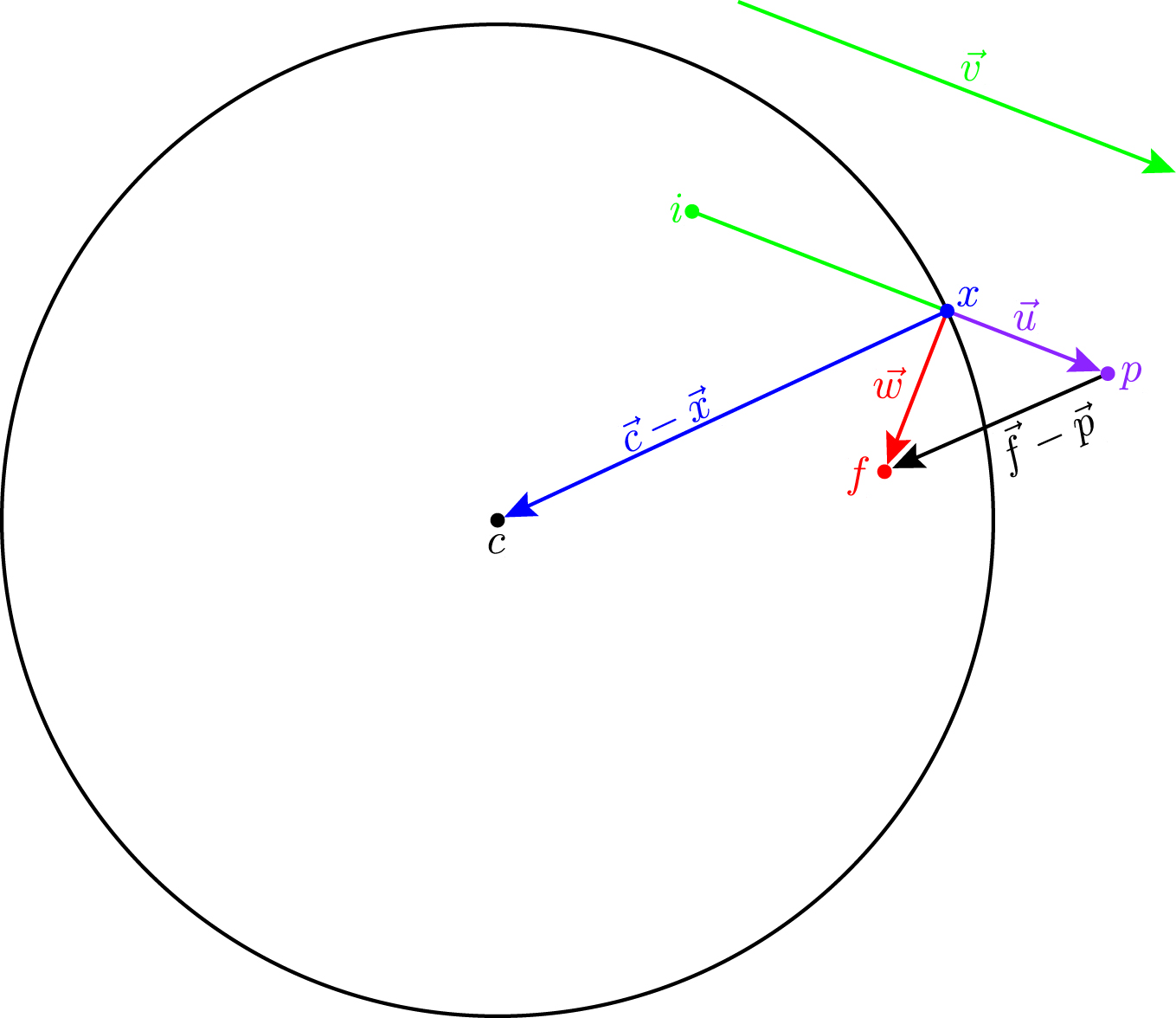


**Figure S2: Important Vectors for Reflecting Inside Actin Island**

Here, we diagram an example of a ‘bound’ particle being reflected back inside an actin island while diffusing during a single time step. Initially the particle is at point $i$; after freely diffusing for one time step, the proposed final position is at point $p$. The particle crosses the circular actin island’s boundary at point $x$. For a reflective boundary, the true final position is at point $f$. Below, we outline the mathematics, which we use to calculate $f$ from points $i$, $p$, $c$ and the radius, $r$, of the island.

*Actin Island boundaries: Derivation*

To reflect a particle inside an actin island (which is a circle in our 2D model), we must know the particle’s initial position inside the island, $i$, the proposed position outside the island, $p$, and the center position of the island,$c$.

First, we find the intersection point (denoted $x$) between the attempted trajectory ($v=p-i$) and the circle. The attempted trajectory defines a line which can be written $i+tv$.

$x=i+t_{x}v$ (S1)

Because $x$ lies on the boundary, we know the following constraint: $\left( x-c \right)\cdot\left( x-c \right)=r^{2}$, where $r$ is the radius of the island. After substituting S1 into the constraint (see S2), we solve for $t_{x}$ (S3).

$\left( i+t_{x}v-c \right)\cdot\left( i+t_{x}v-c \right)=r^{2}$ (S2)

$t_{x}=\frac{v\cdot\left( c-i \right) + \sqrt{Det}}{\left\| v \right\|^{2}}$ (S3.1)

$Det=\left( v\cdot\left( c-i \right) \right)^{2}+\left( \left\| v \right\|^{2} \right)\left( r^{2}-\left\| c-i \right\|^{2} \right)$ (S3.2)

Hence, numerically, $x$ is found by substituting known values into S3 then S1.

The vector $u$ (S4) is the erroneous portion of the particle’s trajectory; that is, the portion in which the particle traveled outside the actin island. To correct the trajectory and reflect the particle back inside the island, we reverse the radial component of $u$. The vector $w$ is the corrected continuation of the trajectory. The magnitudes of $u$ and $w$ are equivalent. They differ by twice the radial component of $u$ (S5). We denote the direction pointing towards the center of the island as $\hat{\left( c-x \right)}=\frac{c-x}{\left\| c-x \right\|}=\frac{c-x}{r}$.

$u=p-x$ (S4)

$w-u=2\left( - \left( u\cdot\hat{\left( c-x \right)} \right) \right)\hat{\left( c-x \right)}$ (S5.1)

$w-u=\left( \frac{-2}{r^{2}} \right)\left( u\cdot\left( c-x \right) \right)\left( c-x \right)$ (S5.2)

And, because $w-u=f-p$, we can calculate $f$ from S5.2.

$f=p-\left( \frac{2}{r^{2}} \right)\left( u\cdot\left( c-x \right) \right)\left( c-x \right)$ (S6)

Numerically, we calculate $x$ from S3 and S1 using known values for $i$, $p$,$c$ and $r$. We then calculate $u$ from S4. To finish, we find the final reflected position, $f$, by solving S6.

*Actin Island boundaries: Implementation*

We name the variables as $i_{x}$ and $i_{y}$ for the *x* and *y* components of the vector $i$. This naming strategy is used throughout the pseudo-code below. Let $\left( i_{x},i_{y} \right)$ be the initial position of the bound particle.

bool **outside** = true (1)

while ( **outside** )

$\left[ \begin{matrix} p_{x} \\ p_{y} \end{matrix} \right]=\left[ \begin{matrix} i_{x} \\ i_{y} \end{matrix} \right]+\left[ \begin{matrix} W_{1} \\ W_{2} \end{matrix} \right]\sqrt{2D_{bound}\Delta t}$ //Diffuse the ‘bound’ particle (2)

**outside** = $\left( p_{x}-c_{x} \right)^{2}+\left( p_{y}-c_{y} \right)^{2}\geq r^{2}$ //check if outside island (3)

if ( **outside** )

$magv2=\left( p_{x}-i_{x} \right)^{2}+\left( p_{y}-i_{y} \right)^{2}$ // ||v||^2 (4)

$vdotci=\left( p_{x}-i_{x} \right)\left( c_{x}-i_{x} \right)+\left( p_{y}-i_{y} \right)\left( c_{y}-i_{y} \right)$ // v DOT (c – i) (5)

$Det=\left( r^{2} \right)-\left( \left( c_{x}-i_{x} \right)^{2}+\left( c_{y}-i_{y} \right)^{2} \right)$ (6)

$Det=\left( vdotci \right)^{2}+\left( magv2 \right)\left( Det \right)$ //calculate Determinant. See (S3.2) (7)

$tx=\frac{vdotci + \sqrt{Det}}{magv2}$ //calculate tx. See (S3.1) (8)

$\left[ \begin{matrix} x_{x} \\ x_{y} \end{matrix} \right]=\left[ \begin{matrix} i_{x} \\ i_{y} \end{matrix} \right]+tx*\left[ \begin{matrix} p_{x}-i_{x} \\ p_{y}-i_{y} \end{matrix} \right]$ //calculate vector x. See (S1) (9)

$\left[ \begin{matrix} u_{x} \\ u_{y} \end{matrix} \right]=\left[ \begin{matrix} p_{x} \\ p_{y} \end{matrix} \right]-\left[ \begin{matrix} x_{x} \\ x_{y} \end{matrix} \right]$ //calculate vector u. See (S4) (10)

$PF=\frac{2}{r^{2}}*\left( \left( u_{x} \right)\left( c_{x}-x_{x} \right)+\left( u_{y} \right)\left( c_{y}-x_{y} \right) \right)$ (11)

$\left[ \begin{matrix} f_{x} \\ f_{y} \end{matrix} \right]=\left[ \begin{matrix} p_{x} \\ p_{y} \end{matrix} \right]-PF*\left[ \begin{matrix} c_{x}-x_{x} \\ c_{y}-x_{y} \end{matrix} \right]$ //calculate Final Position. See (S6) (12)

**outside** = $\left( f_{x}-c_{x} \right)^{2}+\left( f_{y}-c_{y} \right)^{2}\geq r^{2}$ //check if outside island (13)

else {}

end

**Box 3: Diffusing and Reflecting Bound Particles**

We diffuse a ‘bound’ particle within the circular actin island. The particle is allowed to diffuse freely in space (line 2. If the particle lands on the edge of or outside the island (line 3), then we reflect the particle back inside (lines 4 – 12). To catch any pathological cases, arising from numerical error & computational limitations, we re-check if the particle is completely inside (line 13). In some rare cases, this re-check will fail; that is, even after reflecting the particle, the new position will still be on the edge of or outside the island. For those scenarios, we repeat the entire process (from line 2) and calculate a different diffusion step. We find these pathological cases occur when the starting position is close (within the numerical error) to the island boundary. By the end of these calculations, the bound particle has safely & correctly diffused for one time step. This algorithm is an expansion of lines 7 & 8 in Box 1.

**Calculating Reaction Probabilities**

Lines 5 & 9 in Box 1 mark the decision for a molecule to react: either bind (line 5) or unbind (line 9). This decision to react is solely determined by probabilities of reaction. We must calculate these probabilities such that the reactions are simulated in a biophysically relevant regime. Our proposed model for the diffusion of Rac1 in the cell does not (nor is it necessary to) speculate on exact values of the reaction rates for the different actin islands. Instead, our model solely proposes different binding affinities in each island. Below, we derive the relationship between the reaction rates of each island and the probabilities of reaction.

We write out the Master Equation for the binding reaction on the $i^{th}$ island: let $A$ denote the number of ‘unbound’ Rac1 throughout the cell and $A_{i}^{*}$ denote the number of ‘bound’ Rac1 in the $i^{th}$ island.

$\dot{A_{i}^{*}}=\left( k_{\mathrm{on}}^{i}\cdot\frac{1}{V} \right)A-\left( k_{\mathrm{off}}^{i} \right)A_{i}^{*}$ (S7)

Here, $k_{\mathrm{on}}^{i}$ and $k_{\mathrm{off}}^{i}$ are the reaction rates for the $i^{th}$ island, and $V$ is the volume of the cell (we set the cell thickness to $1\mu m$). The unbinding term, $\left( k_{\mathrm{off}}^{i} \right)A_{i}^{*}$, describes the number of molecules which will unbind from the $i^{th}$ island during the infinitesimal time $dt$. The unbinding term is derived from multiplying (a) the off rate by (b) the number of molecules eligible to unbind: $A_{i}^{*}$. The binding term, $\left( k_{\mathrm{on}}^{i}\cdot\frac{1}{V} \right)A$, describes the number of molecules that will bind to the $i^{th}$ island during the infinitesimal time $dt$. The binding term is derived from multiplying (a) the on-rate by (b) the “concentration” of the island and by (c) the number of molecules eligible to bind to the island.

$\left( k_{\mathrm{on}}^{i} \right)\left( \frac{1}{v_{i}} \right)\left( \frac{v_{i}}{V}A \right)=\left( k_{\mathrm{on}}\cdot\frac{1}{V} \right)A$ (S8)

Here, $v_{i}$ is the volume of the $i^{th}$ island; hence, $\frac{1}{v_{i}}$ is the “concentration” (since the unit for concentration is inverse volume) of actin. The number of molecules eligible to bind is the number of unbound molecules located inside the island; this value is equal to the total number of unbound molecules, $A$, scaled by the probability of being located in the $i^{th}$ island: $\frac{v_{i}}{V}$.

At equilibrium, the number of binding and unbinding events are balanced; we set Eqn. S7 equal to $0$ and solve for the $K_{D}$.

$K_{D}^{i}=\frac{k_{\mathrm{off}}^{i}}{k_{\mathrm{on}}^{i}}=\frac{A}{A_{i}^{*}} \frac{1}{V}$ (S9)

Instead of being concerned with actual counts of bound, $A_{i}^{*}$, and unbound, $A$, molecules, we may rewrite the right hand side in terms of the fraction of the entire population: multiply by $\frac{A_{Tot}}{A_{Tot}}$. We denote the fraction of molecules bound to the $i^{th}$ island as $\frac{A_{i}^{*}}{A_{Tot}}=f_{i}$. We write the fraction of unbound molecules as $\frac{A}{A_{Tot}}=1-\sum f_{i}=1-F$, where $F$ is the total fraction of molecules bound in all islands.

$\frac{k_{\mathrm{off}}^{i}}{k_{\mathrm{on}}^{i}}=\frac{1}{V}\frac{A}{A_{i}^{*}}\cdot\frac{A_{Tot}}{A_{Tot}}=\frac{1-F}{f_{i}} \frac{1}{V}$ (S10)

We determine the probability a molecule reacts (both binding and unbinding processes) in a time $\Delta t$. We choose a sufficiently small time step ($1\mu s$) such that we may assume a single molecule can perform only one reaction (bind or unbind) during that time step. Hence, we may separately solve the differential equations describing each reaction (i.e. each term in Eqn. S7).

The probability a molecule unbinds during a time step is equal to $\frac{\# molecules that unbind in \Delta t}{\# bound molecules at time t}=\frac{A_{i}^{*}\left( t \right)-A_{i}^{*}\left( t+\Delta t \right)}{A_{i}^{*}\left( t \right)}$. Where $A_{i}^{*}\left( t \right)$ is the number of molecules bound to the $i^{th}$ island as a function of time. We choose a small time step such that only the $i^{th}$ island unbinds.

$\dot{A_{i}^{*}}=-k_{\mathrm{off}}A_{i}^{*}$ (S11)

$A_{i}^{*}\left( t \right)=Ce^{-k_{\mathrm{off}} \cdot t}$ (S12)

$P_{unbind}=\frac{A_{i}^{*}\left( t \right)-A_{i}^{*}\left( t+\Delta t \right)}{A_{i}^{*}\left( t \right)}=1-\frac{A_{i}^{*}\left( t+\Delta t \right)}{A_{i}^{*}\left( t \right)}=1-\frac{Ce^{-k_{\mathrm{off}} \cdot\left( t+\Delta t \right)}}{Ce^{-k_{\mathrm{off}} \cdot t}}=1-e^{-k_{\mathrm{off}} \cdot\Delta t}$

$\boxed{P_{unbind}=1-e^{-k_{\mathrm{off}} \cdot\Delta t}}$ (S13)

The probability any unbound molecule binds to the $i^{th}$ island during a time step is equal to $\frac{\# molecules that bind in \Delta t}{\# unbound molecules at time t}=\frac{A\left( t \right)-A\left( t+\Delta t \right)}{A\left( t \right)}$. Where $A\left( t \right)$ is the number of unbound molecules as a function of time. We choose a small time step such that only the $i^{th}$ island binds.

$\dot{A}=-k_{\mathrm{on}}^{i}\cdot\frac{1}{V} A$ (S14)

$A\left( t \right)=Ce^{-k_{\mathrm{on}}^{i} \cdot\frac{1}{V} \cdot t}$ (S15)

$P_{on}=\frac{A\left( t \right)-A\left( t+\Delta t \right)}{A\left( t \right)}=1-\frac{A\left( t+\Delta t \right)}{A\left( t \right)}=1-\frac{Ce^{-k_{\mathrm{on}}^{i} \cdot\frac{1}{V} \cdot\left( t+\Delta t \right)}}{Ce^{-k_{\mathrm{on}}^{i} \cdot\frac{1}{V} \cdot t}}=1-e^{-k_{\mathrm{on}}^{i} \cdot\frac{1}{V} \cdot\Delta t}$ (S16)

Equation S16 describes the probability that *any* unbound molecule will bind to the $i^{th}$ island. We rewrite this overall probability as 1) the probability an unbound molecule is located inside the $i^{th}$ island and 2) the probability that same molecule binds/reacts (Eqn S17).

$P_{on}=P_{Isl}P_{bind}=\left( \frac{v_{i}}{V} \right)P_{bind}$ (S17)

Hence, the probability that an unbound molecule located inside an actin island binds during a time step $\Delta t$ is given by Eqn. S18.

$\boxed{P_{bind}=\left( \frac{V}{v_{i}} \right)\left( 1-e^{-k_{\mathrm{on}}^{i} \cdot\frac{1}{V} \cdot\Delta t} \right)}$ (S18)

The exponentials in Eqns. S13 & S18 can be simplified by using the first two terms of its Taylor Expansion. These two expressions for the probability of unbinding and binding can then be written as:

$P_{unbind}=k_{\mathrm{off}} \cdot\Delta t$ (S19)

$P_{bind}=k_{\mathrm{on}} \cdot\frac{1}{v_{i}}\cdot\Delta t$ (S20)

We combine Eqn S10, S19 & S20 to get three equal expressions for the $K_{D}$ relating the reaction rates, fraction bound in each island and the probabilities of reacting.

$\boxed{K_{D}^{i} = \frac{k_{\mathrm{off}}^{i}}{k_{\mathrm{on}}^{i}} = \frac{1-F}{f_{i}} \frac{1}{V} = \frac{P_{unbind}}{P_{bind}} \frac{1}{v_{i}}}$ (S21)

In every simulation, we customize the affinities for each island; that is, we choose the set of $f_{i}$. We perform two simulations for each set of affinities: in one, the off-rate is assigned a value and kept constant while the on-rate is varied; in the other, the on-rate is assigned a value and kept constant while the off-rate is varied. The affinities of the islands and the macro-chemistry are the same for both cases; however, the mechanism is slightly different between the two cases. An off-rate is assigned such that the time scale for unbinding is equal to the time scale for diffusing across an actin island in the ‘bound’ state (see Eqn 7 in Methods). Table S1 summarizes the reaction rates, fraction bound and reaction probabilities for each simulation.

**Full Simulation Parameters**

In all simulations, the volume of the cell, $V$, and the volumes of all islands, $v$, and the time step, $\Delta t$, are all kept constant.

$$V=64{\mu m}^{3}$$

$$v=3.14 {\mu m}^{3}$$

$$\Delta t=1 \mu s$$

We vary the fraction bound in each island. In Table S1, the value for each island is reported as **{A, B, C, D}**, where ‘A’, ‘B’, ‘C’ & ‘D’ are the labels for each island as shown in Fig 5.

| **Simulation**  **Set-up** | $\vec{\boldsymbol{f}}$ | $\vec{\boldsymbol{k}_{\mathbf{off}}}$ $\left( \frac{\boldsymbol{1}}{\boldsymbol{s}} \right)$ | $\vec{\boldsymbol{k}_{\mathbf{on}}}$ $\left( \boldsymbol{10}^{\boldsymbol{9}}\frac{\boldsymbol{1}}{\boldsymbol{M\cdot s}} \right)$ | $\vec{\boldsymbol{P}_{\boldsymbol{unbind}}}$ | $\vec{\boldsymbol{P}_{\boldsymbol{bind}}}$ |
| --- | --- | --- | --- | --- | --- |
| Fig 1C & 4B | {0.125, 0.125,  0.125, 0.125} | {0.5, 0.5  0.5, 0.5} | {4.8, 4.8,  4.8, 4.8} | {0.5×10^-6^, 0.5×10^-6^,  0.5×10^-6^, 0.5×10^-6^} | {2.55×10^-6^, 2.55×10^-6^,  2.55×10^-6^, 2.55×10^-6^} |
| Fig 3A | {0.1875, 0.0625,  0.0625, 0.0625} | {0.5, 0.5  0.5, 0.5} | {5.78, 1.927,  1.927, 1.927} | {0.5×10^-6^, 0.5×10^-6^,  0.5×10^-6^, 0.5×10^-6^} | {3.056×10^-6^, 1.019×10^-6^,  1.019×10^-6^, 1.019×10^-6^} |
| Fig 3D | {0.25, 0.1875,  0.125, 0.0625} | {0.5, 0.5  0.5, 0.5} | {13, 9.7,  6.5, 3.36} | {0.5×10^-6^, 0.5×10^-6^,  0.5×10^-6^, 0.5×10^-6^} | {6.79×10^-6^, 5.09×10^-6^,  3.4×10^-6^, 1.7×10^-6^} |
| Fig 3G | {0.0625, 0.125,  0.1875, 0.25} | {0.5, 0.5  0.5, 0.5} | {3.36, 6.5,  9.7, 13} | {0.5×10^-6^, 0.5×10^-6^,  0.5×10^-6^, 0.5×10^-6^} | {1.7×10^-6^, 3.4×10^-6^,  5.09×10^-6^, 6.79×10^-6^} |
| Fig 4D | {0.03125, 0.0625,  0.09375, 0.125} | {0.5, 0.5  0.5, 0.5} | {0.912, 1.75,  2.6, 3.5} | {0.5×10^-6^, 0.5×10^-6^,  0.5×10^-6^, 0.5×10^-6^} | {4.63×10^-7^, 9.26×10^-7^,  1.39×10^-6^, 1.85×10^-6^} |

**Generating Intensity Carpet**

To generate an intensity carpet as done *in vivo* [1–3] for our *in silico* data, we tally the number of molecules in each bin sequentially, measuring for 25µs at a time. If we label the 256 bins from 0 to 255 and let $t$ be the current simulation time (in µs), then the current bin being tallied is $\left( \left( \mathrm{floor}\left( \frac{t}{25} \right) \right)\mathrm{mod} 256 \right)$. The tally is incremented for every particle positioned inside the current bin after diffusing and reacting. For example: for $t\in\left[ 0,24 \right]$, we tally the particles in bin 0; for $t\in\left[ 25,49 \right]$, we tally the particles in bin 1; for $t\in\left[ 6375,6399 \right]$, we tally the particles in bin 255, and for $t\in\left[ 6400,6424 \right]$, we tally the particles in bin 0. We store these tallies as a matrix with 256 columns (one for each bin), and 47000 rows (one for each line scan).

**Hardware Details**

This program was written in CUDA C using the CUDA 4.0 Toolkit. CUDA is a publicly available parallel programming architecture built for Generally Programmable Graphics Processing Units (GPGPUs), specifically, those manufactured by the NVIDIA ® Corporation. Utilizing GPGPUs, we are able to simultaneously access hundreds of computational processors in parallel. Because each particle is independent of all others, we parallelize Box 1 by unfolding the second loop: “Loop for **M** molecules”. That is, we launch one computational thread in the GPU for each particle in our simulation. Each thread performs all necessary calculations for reacting and diffusing (Boxes 1-3) a single particle. All threads and particles are periodically synchronized in time. Every 12.8ms of simulation time, we stop the threads’ calculations and have the CPU download the tallies for the Intensity Carpet to the RAM. Additionally, the CPU downloads & prints to a file the position & state data of all particles. We run our simulations on the Kill Devil Research Computing Cluster at UNC. Specifically, the program is executed on a computing node whose hardware includes a 12-core 2.67 GHz Intel processor, 48 GB of RAM and an NVIDIA M2070 GPGPU.

**Supplemental References**

1. Digman M a, Gratton E (2009) Imaging barriers to diffusion by pair correlation functions. Biophys J 97: 665–673.

2. Hinde E, Cardarelli F (2011) Measuring the flow of molecules in cells. Biophys Rev 3: 119–129.

3. Hinde E, Digman M a, Hahn KM, Gratton E (2013) Millisecond spatiotemporal dynamics of FRET biosensors by the pair correlation function and the phasor approach to FLIM. Proc Natl Acad Sci U S A 110: 135–140.
